# Associations of premenstrual symptoms with daily rumination and perceived stress and the moderating effects of mindfulness facets on symptom cyclicity in premenstrual syndrome

**DOI:** 10.1007/s00737-023-01304-5

**Published:** 2023-03-11

**Authors:** Sibel Nayman, Desiree Tina Konstantinow, Isabelle Florence Schricker, Iris Reinhard, Christine Kuehner

**Affiliations:** 1 Research Group Longitudinal and Intervention Research, Department of Psychiatry and Psychotherapy, Central Institute of Mental Health, University of Heidelberg, Medical Faculty Mannheim, Mannheim, Germany; 2 Department of Biostatistics, Central Institute of Mental Health, University of Heidelberg, Medical Faculty Mannheim, Mannheim, Germany

**Keywords:** Premenstrual symptoms, Menstrual cycle, Mindfulness, Acceptance, Daily diary

## Abstract

Subthreshold premenstrual symptoms can be impairing even if the diagnostic criteria for premenstrual dysphoric disorder (PMDD) are not reached. Previous research suggests shared psychological risk factors without a clear differentiation of premenstrual syndrome (PMS) from PMDD. This study focuses on a sample with a wide range of premenstrual symptoms not reaching PMDD-criteria and aims to investigate within-person associations of premenstrual symptoms with daily rumination and perceived stress during the late luteal phase as well as cycle-phase specific associations of habitual mindfulness including present-moment-awareness and acceptance with premenstrual symptoms and impairment. Fifty-six naturally cycling women with self-reported premenstrual symptoms completed an online diary on premenstrual symptoms, rumination and perceived stress over two consecutive menstrual cycles, and baseline questionnaires on habitual present-moment-awareness and acceptance. Multilevel analyses revealed cycle-related variations in premenstrual symptoms and impairment (all *p*s < .001). Higher within-person levels of core and secondary premenstrual symptoms during the late luteal phase predicted increased daily rumination and perceived stress (all *p*s < .001) and increased somatic symptoms predicted increased rumination (*p* ≤ .018). Higher habitual present-moment-awareness was linked to lower premenstrual symptom and impairment levels toward the late luteal phase whereas higher habitual acceptance was associated with lower premenstrual functional impairment (*p* ≤ .015). Premenstrual symptom increases during the late luteal phase in women with PMS seem to be linked to increased daily rumination and perceived stress. Trait present-moment-awareness and acceptance in turn seem to reflect protective factors against premenstrual distress and may represent useful targets for interventions.

## Introduction

Most women of reproductive age experience premenstrual symptoms in the week before menses (late luteal phase) which remit within a few days after menstruation onset (follicular phase) (Hofmeister and Bodden 2016; Tschudin et al. 2010). About 1.8–5.8% of these women report severe distress or impairment, and meet the diagnostic criteria of premenstrual dysphoric disorder (PMDD) with at least five symptoms, including at least one core affective symptom (American Psychiatric Association [APA] 2013). If the minimum of required criteria is not reached, the symptoms are classified as premenstrual syndrome (PMS), affecting about 20–30% of women (Mattina and Steiner 2020). The International Society for Premenstrual Disorders (ISPMD) considers PMDD and PMS as categories of core premenstrual disorders (Ismaili et al. 2016). In contrast to PMDD, PMS does not have a unique definition with a specified minimum number of symptoms and is not included in DSM-5 as a diagnostic entity. The PMS definition proposed by the American College of Obstetricians and Gynecologists (ACOG) requires at least one affective and one physical premenstrual symptom with functional impairment during the previous three menstrual cycles— confirmed by a prospective symptom diary (ACOG 2014).

Women who fall short of the required number of five DSM-5 criteria for PMDD may nevertheless experience clinical distress and functional impairment in daily life (Yonkers and Simoni 2018). A dichotomous categorization (PMDD vs. non-PMDD) with a minimum of five symptoms possibly fails to consider these women and can lead to an exclusion from adequate treatments (Kadian and O’Brian 2012). Halbreich et al. (2003) estimate that 13–18% of women with PMS seem to display symptoms to a degree that warrants clinical treatment, although failing to reach the required number of symptoms for a PMDD diagnosis. Relatedly, Hartlage et al. (2012) and Schmalenberger et al. (2017) showed that the number of premenstrual symptoms presenting an optimal threshold to predict functional impairment was four instead of the five symptoms as required by APA (2013).

Dimensional diagnostic approaches represent an alternative to the categorical approach to psychopathology (Michelini et al. 2021). Relatedly, underlying mechanisms of premenstrual disorders have been suggested to exist on a continuum across the spectrum of normal functioning and psychopathology (Eisenlohr-Moul 2019). This perspective results in premenstrual spectrum disorders—with PMDD as the most severe manifestation at the upper end of the continuum, PMS as a milder manifestation, and the absence of symptoms at the lowest end (Yonkers and Simoni 2018). In this regard, samples including PMS as subthreshold-cases of PMDD allow to explore potential common mechanisms involved in the development and maintenance of premenstrual disorders. Identified risk factors or processes in these subthreshold-cases may indicate etiological as well as predictive continuity (cf. van Os 2013) and can be targeted to prevent the transition to a full syndrome disorder (e.g., Shankman et al. 2009).

Psychological risk factors for premenstrual disorders have been insufficiently examined. There is initial evidence for a heightened tendency to ruminate and increased levels of perceived stress in women with premenstrual spectrum disorders (Eisenlohr-Moul 2019; Kappen et al. 2022, preprint; Nayman et al. 2022), especially during the late luteal phase (Beddig et al. 2019; Craner et al. 2016). Habitual rumination (Dawson et al. 2018), momentary rumination in daily life (Beddig et al. 2020), and perceived stress (Beddig et al. 2019; Schweizer-Schubert et al. 2021), in turn, seem to be associated with higher premenstrual mood and symptom worsening in women with PMS or PMDD. Less is known about favorable traits such as present-moment-awareness and acceptance as facets of mindfulness, which has been shown to be linked to lower levels of psychopathology (A-Tjak et al. 2014; Tomlinson et al. 2018). Mindfulness involves deliberately paying attention to present-moment experiences with an accepting and non-judgmental attitude (Kabat-Zinn 2003). A nonclinical study showed that habitual mindfulness with the facets of present-moment-awareness, observing, describing and nonreacting was associated with less premenstrual symptoms (Lustyk et al. 2011), and mindfulness-based interventions appear to reduce premenstrual symptom severity in women with premenstrual disorders (Bluth et al. 2015; Mazaheri Asadi et al. 2022; Panahi and Faramarzi 2016). Similarly, habitual acceptance—possibly weakening the dysfunctional link between detrimental experiences and maladaptive forms of coping (Hayes et al. 2012)—was linked to increased perceived ability to cope with PMS (Read et al. 2014). Kleinstäuber et al. (2016), in contrast, identified no effects of habitual acceptance on premenstrual symptom severity. Nonetheless, higher acceptance has been shown to be associated with lower functional impairment despite existing symptoms in individuals with chronic pain (Lami et al. 2018).

A recent ambulatory assessment study (Nayman et al. 2022) with repeated intensive longitudinal measurements showed that higher habitual present-moment-awareness and reappraisal and lower habitual rumination in women with PMDD were generally associated with better mood in daily life. However, affected women with favorable traits showed a stronger mood worsening toward the late luteal phase, thereby resembling those with less favorable traits. Nonetheless, these results do not preclude the potential role of protective psychological factors during the late luteal phase in women with less severe premenstrual disorders.

In sum, research on psychological factors in women with PMS is sparse and limited by the fact that previous studies did not always strictly differ between PMS and PMDD, which in turn can result in inconsistent findings.

The current study takes a comprehensive approach by combining retrospective assessments of psychological traits with prospective assessments of premenstrual symptoms and functional impairment as well as rumination and perceived stress in daily life over two menstrual cycles in a sample of women with a wide range of premenstrual symptoms, who did, however, *not* meet the number of criteria required for PMDD. Thus, in the current study, we first aimed to investigate cycle-related variations in prospectively assessed PMS symptomatology and impairment across the menstrual cycle in women reporting premenstrual symptoms during a retrospective baseline interview. We expected increases in all prospectively assessed outcomes (core, secondary and somatic symptoms, functional impairment) from the follicular to the late luteal phase. Second, we expected that higher increases in PMS severity (core, secondary, and somatic symptoms) in the late luteal phase would be associated with simultaneously increased rumination and perceived stress during daily life. Third, we expected that habitual present-moment-awareness and acceptance would be associated with weaker premenstrual increases in symptoms and impairments toward the late luteal phase. Given the high comorbidity of premenstrual disorders with depressive disorders (Yonkers and McCunn 2007), we further investigated, whether the expected moderator effects would hold when controlling for depressive symptom levels.

## Materials and methods

### Participants and procedure

Sixty-one female students with premenstrual complaints were recruited from the University of Mannheim, Germany, via post prints, mailing lists, and the digital study register SONA of the University of Mannheim. Women were eligible for the study if they reported at least one affective and one somatic premenstrual symptom as well as functional impairment to at least a mild degree on the Premenstrual Symptom Screening Tool (Bentz et al. 2012; see “Baseline Measures” section). Of these women, only those who did not meet the full PMDD criteria (APA 2013) according to the subsequent 2-month symptom diary (see “Online diary” section) were included to focus on subthreshold PMDD cases. In order to ensure that women had a natural and typical menstrual cycle, further inclusion criteria included age between 18 and 40 years, a reported cycle length between 22 and 34 days, and a body mass index between 18 and 35. Women were ineligible if they had been pregnant or lactating within the last 6 months, had taken hormonal contraceptives during the last 3 months, had a history of ovariectomy, a lifetime diagnosis of bipolar or psychotic disorder, or current substance dependence. Of the 61 recruited women, five met the PMDD criteria (APA 2013) according to the subsequent two-month symptom diary and were thus excluded from the analyses. The final sample consisted of *N* = 56 women. All participants gave written informed consent and were compensated with 50€ or study credit points for their participation.

During an initial telephone-screening, participants provided information on demographics, cycle-related aspects, medical history, and psychiatric comorbidities (based on a checklist for DSM-IV criteria). Preliminarily eligible participants were then invited to the in-person-session at the Central Institute of Mental Health, Mannheim for the assessment of baseline measures of clinical and psychological trait variables (see “Baseline Measures” section), and the introduction to the daily diaries. Daily diaries started on the evening of the in-person-session. Participants rated their symptoms and daily experiences once per day in the evening using a link to the online server of SoCi Survey (www.socisurvey.de) over two menstrual cycles. For the current analyses, data of the late luteal and follicular phase were included. The late luteal phase covered the 7 days before menstruation onset (cycle days − 7 to − 1), whereas the follicular phase covered days 4 to 10 of the menstrual cycle, with day 1 presenting menstruation onset (for a similar approach see Schmalenberger et al. 2017).

### Baseline measures

#### Retrospective screening of premenstrual symptoms

The German version of the 19-item *Premenstrual Symptoms Screening Tool* (PSST; Bentz et al. 2012) was used to screen for premenstrual symptoms and functional impairment on a 4-point-scale (0 = “*not at all*”, 1 = “*mild*”, 2 = “*moderate*”, 3 = “*severe*”; Cronbach’s *α* = 0.903^1^).

#### Depressive symptoms

The German version of the 9-item subscale *PHQ-9* of the Patient-Health-Questionnaire (PHQ; Löwe et al. 2002) was administered to assess depression symptom severity during the previous 2 weeks on a 4-point-Likert scale (Cronbach’s *α* = 0.784^1^).

#### Present-moment-awareness

Participants’ habitual tendency to be attentive and aware of present-moment experiences as one of the facets of the construct of mindfulness was measured using the German version of the 15-item Mindfulness Attention Awareness scale (MAAS; Brown and Ryan 2003). The items were rated on a 6-point Likert scale, with higher scores indicating greater present-moment-awareness (Cronbach’s *α* = 0.849^1^).

#### Acceptance

Trait acceptance as a further facet of the construct of mindfulness was assessed with the respective 3-item subscale (“When I cannot change something, I accept the situation as it is”, “I am able to tolerate and endure uncomfortable situations”, “I am able to accept things as they are”) of the German version of the Heidelberg Form of Emotion Regulation Strategies (HFERST; Izadpanah et al. 2019). Items were rated on a 5-point-scale (Cronbach’s *α* = 0.594^1^).

## Online diary

### Daily premenstrual symptoms

In the first part of the online diary, participants provided self-reports on current premenstrual symptoms and potential functional impairments using the validated 30-item Questionnaire for the Screening of Premenstrual Symptoms (Ditzen et al. 2011) on a daily basis over two menstrual cycles. The questionnaire was validated as both a retrospective questionnaire and a prospective symptom diary (Ditzen et al. 2011; Janda et al. 2017). The first 27 items covered premenstrual symptoms, which were categorically summed into core symptoms (items 1–11; affective lability, irritability, depressed mood, anxiety/tension), secondary symptoms (items 12–19 additional psychological/behavioral symptoms) and somatic symptoms (items 20–27). Three additional items assessed functional impairment. All items were rated on a 4-point Likert-scale ranging from 0 (“*not true at all*”) to 3 (“*absolutely true*”). Based on the scoring approach by Janda et al. (2017), only items which were rated at least ≥ 2 over two consecutive days in the late luteal and a maximum of ≤ 1 in the follicular phase, were considered as “marked.” A DSM-5 PMDD symptom was considered present if one of the corresponding items was rated as “marked” for at least 2 days. Women were excluded from the study if they showed at least five out of eleven symptoms including one affective symptom and functional impairment during the two menstrual cycles and thus met the DSM-5 criteria for PMDD (APA 2013).

#### Daily rumination and perceived stress

The second part of the daily diary included three additional items for the assessment of further facets of daily life experiences including rumination (2 items: “*Today, I was stuck on negative thoughts and could not disengage from them*”, “*Today*, *I ruminated*”) and perceived stress (1 item: “*Today*, *I had a stressful day*”) on a 7-point Likert scale.

### Statistical analysis

Given that daily assessments (level 1) were nested within participants (level 2), we fit multilevel models (MLM) estimating random intercept models with restricted maximum likelihood estimation (REML). All statistical analyses were performed in IBM SPSS Statistics Version 28 with the significance level set at α = 0.05. This value was not adjusted for multiple testing as the tests were based on preplanned hypotheses (Armstrong 2014).

First, cycle-phase-specific variations in daily ratings of premenstrual symptom clusters (core, psychological secondary and somatic symptoms) and functional impairment were estimated in four separate random intercept models. These models included the factor cycle phase (late luteal vs. follicular phase) and the continuous covariate cycle day (1–7) as main effects as well as an interaction term between the two, with premenstrual symptom clusters and functional impairment as outcome variables. Within-person associations of each premenstrual symptom cluster (level 1 predictors) with further facets of daily life experiences (level 1 outcomes: rumination, perceived stress) during the late luteal phase were investigated by separate random intercept models with the data of the late luteal phase. For these analyses, level-1 predictors were decomposed into within- and between-person components by late-luteal-phase-specific person-mean- and grand-mean-centering. Within-person effects reflect how variations of daily experiences within a person are associated with variations in subjective or physiological outcomes (e.g., how intraindividual variations in daily premenstrual symptoms affect daily rumination within a person; Myin-Germeys and Kuppens 2022). Between-person effects reflect differences between individuals (e.g., how women with higher premenstrual symptoms differ from those with lower premenstrual symptoms with regard to daily rumination).

Finally, moderator effects of habitual present-moment-awareness and acceptance (level 2) on the associations of cycle phase with each premenstrual symptom cluster and functional impairment were tested with a set of additional models. These models included interaction terms of psychological traits by cycle phase. In a further set of interaction analyses, depressive symptom severity (PHQ-9) was added to the models as a level 2 covariate. Here, all level-2 variables were grand-mean centered, allowing the estimation of between-person effects.

According to the summary-statistics-based power analysis for mixed-effects models (Murayama et al. 2022), our sample size of *N* = 56 was sufficient to achieve 80% power to detect small to medium effect sizes with α set at 0.05.

## Results

Table 1 presents demographic, psychological and clinical characteristics of the current sample (*N* = 56). Overall, 1497 out of a total of 1568 daily assessments (56 participants × 7 assessment days × 2 cycle phases × 2 menstrual cycles) were recorded, corresponding to a high compliance rate of 95.5% (cf. Wrzus and Neubauer 2022). The intra-class correlations (ICC) of daily records of symptoms (ICC_core_ = 0.24, ICC_secondary_ = 0.32, ICC_somatic_ = 0.32), functional impairment (ICC_functional_impairment_ = 0.33), and further daily life experiences (ICC_rumination_ = 0.35, ICC_perceived_stress_ = 0.24) indicated that a considerable amount of variance in state outcomes is attributable to within-person differences.

**Table 1:** Demographics and psychological measures

	*M* (*SD*)	Range
Demographic Variables		
Age	22.1 (3.3)	18–33
Menstrual cycle during the study		
Length of cycle 1	29.0 (4.3)	20–42
Length of cycle 2	28.4 (3.2)	19–39
Baseline measures		
MAAS	62.3 (10.4)	35–83
HFERST_A	9.4 (2.1)	3–13
PHQ-9	7.1 (3.9)	1–20
PSST	24.63 (10.38)	7–47
Daily measures		
Daily rumination	6.10 (3.14)	2–14
Daily perceived stress	3.41 (1.74)	1–7

For all baseline measures, sum scores were calculated. MAAS, Mindfulness Attention Awareness scale; HFERST_A, Heidelberg Form of Emotion Regulation Strategies – Acceptance Subscale; PHQ-9, Patient-Health-Questionnaire-9; PSST, Premenstrual Symptoms Screening Tool

### Cycle-phase-specific variations of premenstrual symptoms

**Fig. 1:**
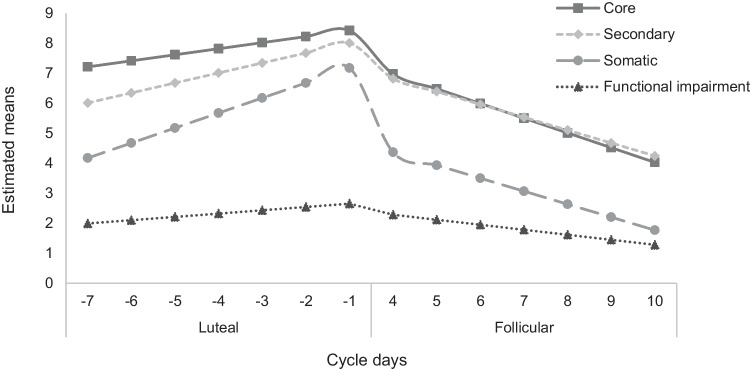
Cycle-related variations of core, secondary and somatic symptoms and functional impairment. Four separate random intercept models of cycle day * cycle phase on each symptom cluster and functional impairment were estimated

Random intercept models revealed significant interaction effects of cycle phase by cycle day on daily symptom ratings. As illustrated in Fig. 1, core (*F*(1,1439) = 17.84, *p* < 0.001), secondary (*F*(1, 1439) = 40.44, *p* < 0.001), and somatic symptoms (*F*(1,1439) = 103.12, *p* < 0.001) as well as perceived functional impairment (*F*(1,1439) = 31.61, *p* < 0.001) increased in the late luteal phase and decreased in the in the follicular phase.

### Effects of premenstrual symptoms on daily rumination and perceived stress

Next, we examined the associations of daily premenstrual symptoms with daily rumination and perceived stress in the late luteal phase. Results are presented in Table 2. Random intercept models revealed that higher within-person levels of core and secondary symptoms were associated with increased daily rumination (all *p*s < 0.001) and perceived stress (all *p*s ≤ 0.018) in the late luteal phase. Higher within-person levels of somatic symptoms predicted increased daily rumination (*p* < 0.001), with no effects on perceived stress in the late luteal phase (*p* = 0.111).

**Table 2:** Main effects of premenstrual symptom clusters on daily rumination and perceived stress during the late luteal phase

	Rumination				Perceived stress			
Predictor	*B (SE)*	*df*	*t*	*p*	*B (SE)*	*df*	*t*	*p*
Core symptoms								
Intercept	1.91 (0.18)	55	10.85	< .001	2.84 (0.22)	53	12.69	< .001
Core symptoms (B-S)	0.16 (0.02)	56	8.34	< .001	0.06 (0.02)	55	2.39	.020
Core symptoms (W-S)	0.12 (0.01)	675	19.86	< .001	0.05 (0.01)	674	6.02	< .001
Secondary symptoms								
Intercept	1.88 (0.22)	54	8.77	< .001	2.57 (0.23)	53	11.11	< .001
Secondary symptoms (B-S)	0.18 (0.03)	55	6.72	< .001	0.10 (0.03)	53	3.67	< .001
Secondary symptoms (W-S)	0.10 (0.01)	676	10.46	< .001	0.03 (0.01)	674	2.36	.018
Somatic symptoms								
Intercept	2.42 (0.24)	54	10.02	< .001	3.11 (0.24)	53	13.22	< .001
Somatic symptoms (B-S)	0.13 (0.04)	54	3.55	< .001	0.03 (0.03)	53	0.88	.383
Somatic symptoms (W-S)	0.08 (0.01)	676	6.32	< .001	0.03 (0.02)	675	1.60	.111

All models include random intercepts at level 2. *B*-*S* between-person (grand mean centered), *W*-*S* within-person (person mean centered), *B* unstandardized coefficient, *SE* standard error, *df* degrees of freedom

### Interaction effects of psychological traits and cycle phase

In a next step, we investigated possible moderator effects of habitual present-moment-awareness and acceptance on cycle-phase-specific levels of premenstrual symptoms. Results are presented in Table 3. Trait present-moment-awareness significantly moderated the associations of cycle phase with core, secondary and somatic symptoms, and functional impairment (all *p*s ≤ 0.015). The interaction effect of trait acceptance and cycle phase was significant for functional impairment (*p* = 0.015), but not for premenstrual symptom clusters (*p* > 0.05).

**Table 3:** Interaction effects of psychological traits and cycle phase on daily premenstrual symptoms and functional impairment

	Core symptoms			Secondary symptoms			Somatic symptoms			Functional impairment		
Predictor	*df*	*F*	*p*	*df*	*F*	*p*	*df*	*F*	*p*	*df*	*F*	*p*
MAAS x Cycle phase^a^	1, 1440	5.94	.015	1, 1440	12.80	< .001	1, 1440	25.53	< .001	1, 1440	9.63	.002
HFERST_A x Cycle phase^a^	1, 1441	1.25	.264	1, 1440	2.54	.112	1, 1440	0.04	.845	1, 1440	5.92	.015

All models include random intercepts at level 2 and fixed effects of cycle phase and respective psychological traits. *MAAS* Mindfulness Attention Awareness scale, *HFERST*_*A* Heidelberg Form of Emotion Regulation Strategies– Acceptance Subscale, *df* degrees of freedom

^a^Reference category: follicular phase

**Fig. 2:**
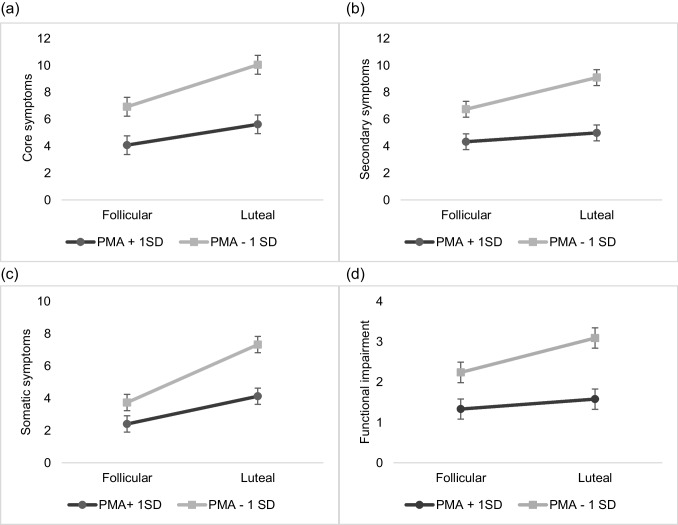
Interaction effects of trait present-moment-awareness (PMA) and cycle phase on premenstrual **a** core, **b** secondary and **c** somatic symptoms, and **d** functional impairment. Estimated mean values of premenstrual symptoms and functional impairment per menstrual cycle phase for low and high scores on PMA (Mindfulness Attention Awareness scale; M ± 1SD)

**Fig. 3:**
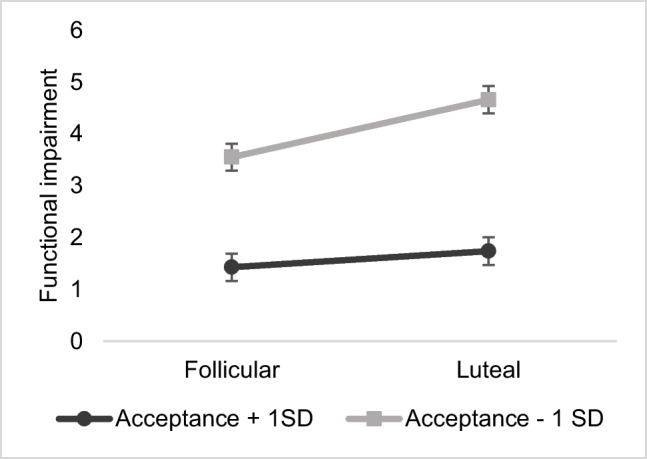
Interaction effect of trait acceptance and cycle phase on premenstrual functional impairment. Estimated mean values of premenstrual functional impairment per menstrual cycle phase for low and high scores on acceptance (Heidelberg Form of Emotion Regulation Strategies–Acceptance Subscale; M ± 1SD)

As depicted in Fig. 2 for illustration purposes, higher present-moment-awareness was linked to lower levels in core, secondary, and somatic premenstrual symptoms as well as to lower levels in functional impairment toward the late luteal phase. Acceptance, in turn, was only associated with lower increases in functional impairment in the late luteal phase (Fig. 3).

The severity of depressive symptoms (PHQ-9) as a covariate did not change the results of any reported trait × cycle phase interaction effects (all interaction *p*s ≤ 0.016).

## Discussion

The present study aimed to investigate the cyclical variation of premenstrual symptoms and functional impairment in a sample of women with a wide range of premenstrual symptoms, excluding cases with PMDD. Additional goals were to uncover within-person associations of premenstrual symptoms with further facets of daily life experiences (i.e., rumination and perceived stress) during the late luteal phase, as well as to explore associations of habitual present-moment-awareness and acceptance with premenstrual symptoms and impairment over the cycle. As expected, both premenstrual symptoms and impairment showed a cyclical pattern by increasing toward the late luteal phase and decreasing during the follicular phase after menstruation onset. In particular, the demonstrated premenstrual increase in functional impairment indicates that premenstrual symptoms, which do not reach the DSM-5 criteria for PMDD (APA 2013), may also interfere with daily activities during the late luteal phase and thus should be considered clinically relevant if premenstrual impairment is present (cf. Halbreich et al. 2003; Schmalenberger et al. 2017).

Higher within-person levels of premenstrual symptoms were associated with increased daily rumination and perceived stress during the late luteal phase. These results coincide with previous findings (Beddig et al. 2019; Craner et al. 2016) and point to a broader impact of premenstrual symptoms on daily life experiences, even in women with subthreshold PMDD symptoms. Premenstrual increases in daily rumination and perceived stress, in turn, have been shown to impede premenstrual mood (Beddig et al. 2019, 2020). Consequently, premenstrual symptoms, perceived stress, and daily rumination may reciprocally reinforce each other and result in a vicious cycle, possibly maintaining and worsening premenstrual symptomatology in the long-term. Thus, subthreshold conditions of PMDD might represent an important target group for interventions to prevent symptom chronification or transition to PMDD.

Trait present-moment-awareness was associated with weaker increases in core, secondary, and somatic premenstrual symptoms and impairment toward the late luteal phase. These results extend limited findings from previous cross-sectional (Lustyk et al. 2011) and treatment studies (e.g. Bluth et al. 2015), indicating protective effects of habitual mindfulness in women with premenstrual disorders. In contrast, a recent Ambulatory Assessment study on women with PMDD showed that whereas trait present-moment-awareness was generally linked to better mood, women with higher levels of present-moment-awareness paradoxically showed a stronger mood worsening toward the late luteal phase, such that their premenstrual mood levels converged with those of women with lower present-moment-awareness (Nayman et al. 2022). These apparently opposing patterns in the associations of trait present-moment-awareness and premenstrual mood in women with PMDD and those with less severe premenstrual symptoms might reflect a differential role of transdiagnostic psychological factors such as present-moment-awareness for these conditions. This may also imply that addressing present-moment-awareness could represent a psychotherapy target especially for less severe variants of premenstrual disorders such as PMS. However, this conclusion is clearly speculative and requires further research using intervention studies with randomized controlled trials.

In line with Kleinstäuber et al. (2016), habitual acceptance was not linked to premenstrual symptom severity in the present study. However, women with higher acceptance scores showed lower premenstrual functional impairment such that higher acceptance seems to be linked to lower symptom interference with daily activities despite existing symptoms, as already shown for other chronic conditions such as chronic pain (e.g., Lami et al. 2018). Similarly, Read et al. (2014) assume that women with PMS who accept their symptoms may be more likely to perceive a higher ability to cope with premenstrual symptoms. Thus, the attitude of accepting distressing experiences such as premenstrual symptoms may put individuals into a more active and flexible state to handle daily life activities in spite of symptoms (cf. Hayes et al. 2012).

### Strengths and limitations

A major strength of this study is its comprehensive approach combining trait assessments with an online diary to assess different clusters of premenstrual characteristics (i.e., symptoms, impairment, daily rumination, and perceived stress) over two cycles. In the context of research on premenstrual disorders, longitudinal designs with repeated assessments allow to consider cycle-related within-person variations in clinical and psychological characteristics (Bosman et al. 2016; Eisenlohr-Moul 2019). Additionally, the use of an online symptom diary with records of survey dates instead of paper–pencil versions of cycle calendars enabled us to monitor participants’ compliance with diary records.

Some limitations of this study are noteworthy. The sample consisted of university students without psychiatric comorbidities and hormonal contraceptive use, thus possibly limiting the generalizability of the results. Another limitation is the lack of ovulation testing to validate ovulatory cycles. Furthermore, to our knowledge, there are no standardized scales for the daily assessments of rumination and perceived stress, and the current findings should be replicated with standardized scales for daily assessments in future research. In this context, the experience sampling method (ESM) item repository initiative (Kirtley et al. 2022) may contribute to generating reliable short scales in future. Finally, given that this study did not use an experimental design and assessed habitual present-moment-awareness and acceptance only once during the baseline-session without considering the current cycle phase, no conclusions on cycle-phase-specific characteristics and causality of the associations between the respective trait and state variables can be drawn. Moreover, possible overlapping effects of premenstrual symptoms such as concentration difficulty on the measurement of trait of present-moment-awareness cannot be ruled out.

## Conclusions

The present longitudinal study confirms that both premenstrual symptoms and functional impairment increase toward the late luteal phase in women with a broader range of PMS manifestations, and shows that premenstrual symptoms predict increased daily rumination and perceived stress during the late luteal phase. In addition, both habitual present-moment-awareness and acceptance seem to reflect protective factors with regard to premenstrual distress in these conditions and thus might represent useful targets for interventions in women with PMS.

## Data Availability

The dataset generated and analyzed during the current study is available from the corresponding author on reasonable request.

## Footnotes

1 All reported Cronbach’s alpha values (α) in this paper refer to the present sample.
